# Association between smokeless tobacco use and cigarette smoking amount by age

**DOI:** 10.1186/s12889-022-12929-z

**Published:** 2022-03-15

**Authors:** Jin-Won Noh, Min-Hee Kim, Yejin Lee, Young Dae Kwon, Kyoung-Beom Kim, Hae-Jeung Lee, Ki-Bong Yoo

**Affiliations:** 1 Division of Health Administration, College of Software and Digital Healthcare Convergence, Yonsei University, Wonju, 220710 Korea; 2 Department of Physical Therapy, Eulji University, Seongnam, Republic of Korea; 3 Department of Public Health, Graduate School, Korea University, Seoul, 02841 Republic of Korea; 4 Department of Humanities and Social Medicine, College of Medicine and Catholic Institute for Healthcare Management, The Catholic University of Korea, Seoul, Republic of Korea; 5 Department of Health Administration, Dankook University, Yongin, Republic of Korea; 6 Department of Food & Nutrition, Gachon University, Seongnam, Republic of Korea

**Keywords:** Smokeless tobacco, Smoking-cessation, Propensity score matching

## Abstract

**Background:**

The use of smokeless tobacco has increased worldwide among young people. This study aimed to investigate the association between smokeless tobacco use and cigarette smoking amount in adult smoker groups stratified by age.

**Method:**

2013–2015 National Health Interview Survey was used. A total of 19,635 subjects were included in our analysis. Propensity score matching was used to adjust for selection and any other bias. Generalized estimating equation was used to analyze the association between smokeless tobacco use and cigarette smoking amount by age.

**Results:**

All 580 smokeless tobacco users were matched to 2,900 non-smokeless tobacco users. Among those who were aged under 30, smokeless tobacco use was positively associated with the number of cigarettes used per day. Smokeless tobacco users who were aged under 30 and tried quitting smoking used more cigarettes than those who did non-smokeless tobacco users.

**Conclusions:**

The present study revealed that among those who were aged under 30, smokeless tobacco use was positively associated with the number of cigarettes used per day. This study could contribute to understand the behaviors and tendencies of smoking in young adulthood and to establish effective smoking cessation methods for their age.

## Background

Cigarette smoking influences harmfully most organs in the body and is associated with approximately 480,000 US deaths every year [1]. Due to the recognition of the risk of cigarette smoking, the prevalence of cigarette smoking has decreased gradually [2]. Nevertheless, the use of smokeless tobacco has not decreased, but rather increased in the USA and some northern European countries, especially among young people. It is reported that smokeless tobacco products, such as snuff, snus and chewing tobaccos, are highly used in the USA [3], Sweden [4, 5], India [6], and other countries in Southeast Asia [7]. In smokeless tobacco prevalence, the United States had the highest men prevalence rates among men in region of the Americas (7.1%). The Sweden had higher prevalence rates than other European countries regardless of sex (Total: 17.0%, Men: 26.0%, Women: 7.0%) [8]. Smokeless tobacco is consumed through the mouth or nose in the form of chewing, spitting, dipping, or snuffing without burning [9], which is a chemical compound, which includes chemical nicotine and potent tobacco-specific nitrosamines [10]. Smokeless tobacco is a severe risk factor for oral cancer, myocardial infarction, and stroke [11, 12]. Smokeless tobacco use, particularly among young adults, can lead to severe chronic disease burden of cancers and ischemic heart disease, in adulthood [13]. Moreover, smokeless tobacco use should be observed carefully because its use is positively associated with the number of cigarettes used during young adulthood.

The prevalence of smokeless tobacco use is increasing due to the exponential smokeless tobacco marketing expenditures, which increased over 300% from 250.8 million dollars (in 2006) to 759.3 million dollars (in 2016) [14, 15]. Furthermore, young adults have long been tailored to the target of smokeless tobacco marketing [14, 16, 17], and this is likely a major factor in the excessive increase of smokeless tobacco use among young adults aged 18–25, compared to older age groups [14, 18–20]. People initiate smokeless tobacco because they considered these products as less harmful and lower premature mortality than cigarette smoking [21], which are marketed as substitutes for cigarette smoking [1]. However, it was reported that severe dental disease and cardiopulmonary cancer are attributable to smokeless tobacco [22], and the risk of cancer is higher in smokeless tobacco users than in non-users of any form of tobacco [23]. Moreover, previous studies have indicated higher nicotine levels among smokeless tobacco users as compared to cigarette smokers which suggest strong nicotine dependence associated with smokeless tobacco use [24, 25].

Previous studies about smokeless tobacco use and the onset or behavior of cigarette smoking have shown methodological limitations. The characteristics of the comparison group and interested group in previous studies were not similar, due to the view that smokeless tobacco use is just alternative to cigarette smoking, not independent smoke group [26–30]. These studies have limitations because the user group of smokeless tobacco and non-smoker groups was not matched exactly, and therefore, a direct comparison is not appropriate. The risk perceptions of smokeless tobacco have been correlated with the use of those products in adults [31, 32]. Indeed, fewer studies have reported the impact of using smokeless tobacco on frequent and intensive cigarette smoking in terms of age. Moreover, previous studies have reported about the young adult population to understand smoking behavior for tobacco control. However, these reports focused on factors associated with smoking behaviors only in young adults or investigated the differences of characteristics among young adults and other age groups. Thus, the differences within the young adult population are deficient [33].

Therefore, this study aimed to investigate the association between smokeless tobacco use and smoking amount in adult groups stratified by age, using a propensity score matching method and controlling for socioeconomic status. This study considers and analyzes the use of smokeless tobacco as another form of smoking behavior, not the alternatives to cigarette smoking.

## Methods

### Data and study population

This study used data from the National Health Interview Survey (NHIS) conducted in the United States. It was managed by the National Center for Health Statistics in Center for Disease Control and Prevention. The NHIS has been conducted annually since 1963 to monitor information on the general health status in the United States through personal household interviews. It includes socio-economic status, health behaviors, and other various health problems related to the national health objectives. The NHIS is a nationally representative cross-sectional survey based on a multistage clustered area probability sample. Given that data on smokeless tobacco use have been included in the sample adult files of NHIS since 2013, data from the NHIS 2013–2017 were selected for this study. The total populations of sample adult files were 164,696, with 34,557 in 2013, 36,697 in 2014, 33,672 in 2015, 33,028 in 2016, and 26,742 in 2017. Household, person, income files of NHIS were merged into adult files. We excluded non-smokers and past smokers (*n* = 136,644), those who did not mention the number of cigarettes used per day (*n* = 7,242), and those with missing values for smokeless tobacco use (*n* = 972), education level (*n* = 83), marital status (*n* = 29), alcohol consumption (*n* = 79), job status (*n* = 6), or attempt to quit smoking (*n* = 6). Finally, a total of 19,635 subjects were included in our analysis. This study was approved by the Institutional Review Board of Eulji University (EUIRB2018-3).

### Variables

The number of cigarettes used per day was the dependent variable in this study. It was derived from the question, “On an average, how many cigarettes do you now smoke a day?” For the independent variables, we selected factors associated with cigarette smoking and smokeless tobacco use [29, 34, 35]. Age, sex, race, marital status, educational level, household income, job status, alcohol consumption, body mass index (BMI), physical activity, attempt to quit smoking, smokeless tobacco use, and survey year were used in the analyses. Race was categorized as whites, African Americans, or others. Marital status was classified as single, widowed, divorced or separated group or married. Educational level was classified as under high school, high school, or above high school. Alcohol consumption was classified as never, former, or current. Age, household income, and BMI were included as continuous variables. Household income was calculated by dividing the household monthly income by the square root of the household size and log-transformed. Job status was categorized into blue collar workers, unemployed that subjects were looking for work, or all other categories. The frequency of vigorous and light/moderate activity was used as a measure of physical activity. We classified the frequency of physical activity into active, insufficiently active, and inactive. Firstly, minutes of moderate-intensity equivalent activity were calculated by adding the minutes of light/moderate activity and the minutes of vigorous activity. We calculated one minute of vigorous activity as two minutes of light/moderate activity. If minutes of moderate-intensity equivalent activity were 150 min/week or more, the subjects were classified to the active category. If minutes of moderate-intensity equivalent activity were under 150 min/week, the subjects were classified to the insufficiently active category. If minutes of moderate-intensity equivalent activity were reported under 10 min/week, the subjects were classified to the inactive category [36].

Attempt to quit smoking was evaluated using the question, “During the past 12 months, have you stopped smoking for more than one day because you were trying to quit smoking?”. The answer was binary with yes or no. Smokeless tobacco use was identified by the question, “Do you now use smokeless tobacco products every day, some days, or not at all?” If the subjects answered every day or some days, they were classified as smokeless tobacco user. If they answered ‘not at all or rarely’ or ‘had not ever used smokeless tobacco products even once,’ they were classified as non-smokeless tobacco users.

### Statistical analyses

In this study, we employed propensity score matching (PSM) to adjust for selection and any other bias. As NHIS is a complex multistage design survey, we employed DuGoff et al.’s approach to estimate the average treatment effect on the treated [37]. A propensity score was obtained by using binary logistic regression for smokeless tobacco use (yes/no), adjusting for age, sex, race, marital status, educational level, household income, alcohol consumption, BMI, physical activity, attempt to quit smoking, survey year, and sampling weight. After calculating a propensity score for each subject, we used greedy matching method [38]. The case–control matching ratio was 1:5. Standardized differences were calculated for all independent variables except sampling weight before and after matching. The threshold of a standard difference is usually 0.1 to 0.2, which indicates negligible difference in the mean or ratio of covariates between smokeless tobacco users and non-smokeless tobacco users [39, 40]. Association between smokeless tobacco and number of cigarettes used per day was examined by survey Poisson regression in Stata/MP 15.1 with taking into account the complex sampling design using survey weights. For subgroup analyses by age (< 30, 30–44, ≥ 45) and attempt to quit smoking (yes/no), we recalculated all new propensity scores and developed a new matched dataset for each subgroup. All standardized differences of all subgroup were under 0.2. All *p* values were two-sided and considered significant at *p* < 0.05.

### Patient and Public Involvement

No patient involved.

## Results

Table 1 shows the general characteristics of the study participants before and after PSM. All standardized differences were < 0.1 for all matched covariates in all datasets. All 580 smokeless tobacco users were matched to 2,900 non-smokeless tobacco users. Those who used smokeless tobacco used a higher number of cigarettes per day than did non-smokeless tobacco users. The standardized difference in the number of cigarettes used per day was only 0.06 after PSM even though the number of cigarettes used per day was not included when estimating PSM.

**Table 1:** General characteristics of study population before and after propensity score matching

	**Smokeless tobacco use**				**Standardized difference after matching**
	**Before propensity score matching**		**After propensity score matching**		
	**No (*n* = 19,048)**	**Yes (*n* = 587)**	**No (*n* = 2,900)**	**Yes (*n* = 580)**	
Number of cigarettes used per day	13.9 ± 0.1	15.0 ± 0.5	14.4 ± 0.2	15.0 ± 0.5	0.07
Age (year)	46.7 ± 0.2	37.5 ± 0.7	38.3 ± 0.3	37.5 ± 0.7	0.01
Body mass index	29.2 ± 0.1	28.5 ± 0.4	28.4 ± 0.3	28.5 ± 0.4	-0.00
Ln (household income)	10.0 ± 0.0	10.0 ± 0.0	10.1 ± 0.0	10.0 ± 0.0	-0.00
Sex					
Men	9,163 (84.3)	520 (15.7)	2,543 (71.0)	513 (29.0)	0.02
Women	9,885 (93.3)	67 (6.7)	357 (63.5)	67 (36.5)	
Race					
Whites	15,219 (88.3)	525 (11.7)	2,590 (69.1)	520 (30.9)	0.03
African Americans	2,763 (94.3)	35 (5.7)	165 (80.6)	34 (19.4)	
Others	1,066 (93.6)	27 (6.4)	145 (83.0)	26 (17.0)	
Marital status					
Single	6,597 (86.4)	240 (13.6)	1,276 (70.7)	236 (29.3)	0.08
Widowed, divorced or separated	6,409 (88.7)	156 (11.3)	770 (69.6)	153 (30.4)	
Married	6,042 (86.5)	191 (13.5)	854 (66.2)	191 (33.8)	
Education level					
Under high school	864 (86.9)	25 (13.1)	102 (66.9)	25 (33.1)	0.04
High school	9,768 (87.8)	337 (12.2)	1,656 (71.3)	333 (28.7)	
Above high school	8,416 (87.7)	225 (12.3)	1,142 (66.7)	222 (33.3)	
Job status					
All others	11,550 (91.2)	220 (8.8)	1,473 (74.8)	217 (25.2)	0.04
Blue collar workers	6,104 (82.0)	316 (18)	1,193 (60.8)	312 (39.2)	
Unemployed	1,394 (79.5)	51 (20.5)	234 (66.7)	51 (33.3)	
Alcohol consumption					
Never drinkers	1,682 (89.5)	33 (10.5)	126 (63.5)	32 (36.5)	0.06
Former drinkers	3,595 (88.4)	63 (11.6)	289 (65.8)	62 (34.2)	
Current drinkers	13,771 (87.4)	491 (12.6)	2,485 (70.6)	486 (29.4)	
Physical activity					
Inactive	8,906 (88.3)	238 (11.7)	1,167 (70.4)	236 (29.6)	0.01
Insufficient	4,617 (87.9)	122 (12.1)	604 (66.7)	121 (33.3)	
Active	5,525 (86.1)	227 (13.9)	1,129 (70.5)	223 (29.5)	
Tried to quit smoking					
Yes	8,410 (86.6)	287 (13.4)	1,417 (67.9)	285 (32.1)	0.01
No	10,638 (88.2)	300 (11.8)	1,483 (69.9)	295 (30.1)	
Year					
2013	4,548 (85.4)	123 (14.6)	596 (65.8)	121 (34.2)	0.03
2014	3,939 (87.2)	131 (12.8)	683 (70.5)	131 (29.5)	
2015	3,726 (84.5)	114 (15.5)	538 (65.9)	111 (34.1)	
2016	3,904 (88.8)	133 (11.2)	658 (71.6)	131 (28.4)	
2017	2,931 (89.8)	86 (10.2)	425 (71.8)	86 (28.2)	

Values: weighted mean ± standard error or n (weighted %)

**Fig. 1:**
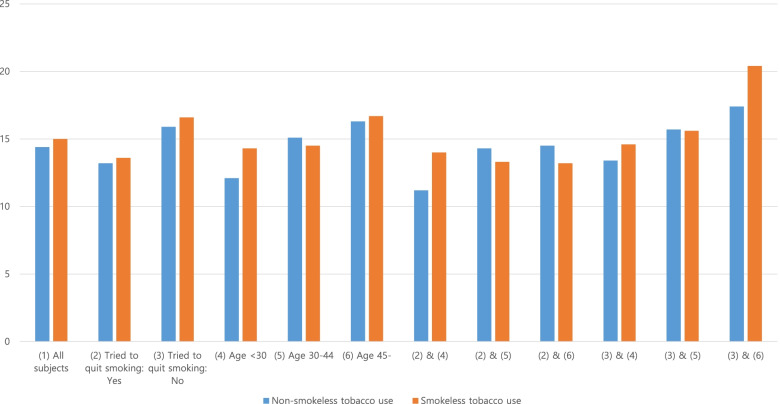
Number of cigarettes used per day for each subgroup

We stratified the study population by age group and whether they had tried to quit smoking. The number of cigarettes used per day in each subgroup is shown in Fig. 1. There was a remarkable difference in those who were aged under 30 and had tried to quit smoking (2&4).

Table 2 shows the results of the survey Poisson regression for all matched subgroup data. For all subjects (1), smokeless tobacco use was not associated with the number of cigarettes used per day. However, among those who were aged under 30 years old (4), smokeless tobacco use was significantly associated with the number of cigarettes used per day. Among those who were aged under 30 and who had tried to quit smoking (2&4), an association was found between smokeless tobacco use and the number of cigarettes used per day.

**Table 2:** Associations of smokeless tobacco use with number of cigarettes used per day

Subgroups	PSM		
	**Coefficient**	***P*-value**	**95% Confidence Interval**
(1) All subjects	0.044	0.229	(-0.028—0.115)
(2) Tried to quit smoking: yes	0.025	0.655	(-0.085—0.135)
(3) Tried to quit smoking: no	0.039	0.477	(-0.069 – 0.148)
(4) Age < 30	0.164	0.015	(0.032 – 0.230)
(5) Age 30–44	-0.044	0.441	(-0.156—0.068)
(6) Age 45-	0.027	0.733	(-0.129 – 0.184)
(2) & (4)	0.230	0.036	(0.015—0.444)
(2) & (5)	-0.073	0.344	(-0.225 – 0.079)
(2) & (6)	-0.094	0.335	(-0.285 – 0.097)
(3) & (4)	0.088	0.457	(-0.145 – 0.321)
(3) & (5)	-0.004	0.948	(-0.132 – 0.123)
(3) & (6)	0.160	0.121	(-0.042—0.362)

PSM: results from propensity score matching with adjusting age, body mass index, ln (household income), sex, race, marital status, education level, job status, alcohol consumption, physical activity, tried to quit smoking, and year. Calculating propensity score and matching were conducted for each subgroup

## Discussion

This study is to investigate the association between smokeless tobacco use and smoking amount in adult groups stratified by age. Among 580 smokeless tobacco users and 2,900 non-smokeless tobacco users, those who were aged under 30 showed significant associations between smokeless tobacco use and the number of cigarettes used per day. The results of this study identified that the use of smokeless tobacco would be associated with the amount of cigarette smoking according to age and attempt to quit smoking. In the ages of 18 to 30 years, smokeless tobacco use was significantly associated with the number of cigarettes used per day. In addition, among those who were 29 years old or less and had tried to quit smoking, an association was found between smokeless tobacco use and the number of cigarettes used per day.

The significant associations between usage of smokeless tobacco product and the number of cigarettes smoked on average day in youth. These results of this study are similar to previous studies that smokeless tobacco is a means to initiate smoking in young adults [26, 29, 34]. It was reported that young adulthood is important to determine whether smoking may be maintained or cease. The cessation of smoking before around the age of 30 years can prevent various harmful effects of smoking, resulting in evident survival rates compared with people who have no experience of smoking [41]. However, the prevalence of using smokeless tobacco was higher among young adults than that among older adults [30]. Furthermore, for young adults, smokeless tobacco was related to the onset of smoking behavior, and it had more impact on young adults [26, 42]. Thus, it is important to identify and analyze the behavior and related factors of smoking in young adulthood according to age for cessation.

Meanwhile, previous research studied that cigarette substitutes, such as e-cigarette, use is strongly associated with current smoking. The use of cigarette substitutes tends to be associated with a lack of obvious quitting intention [33]. Young adults used fewer cigarettes per day than those aged 25 years and older, highly attempted to quit smoking, and had a less health professional talk about smoking [43]. There are only few studies reported that the relation of quit attempt rates between smokeless smokers and not [44]. However, it was reported that many dual users who were cigarette users with smokeless tobacco appeared to use smokeless tobacco for smoking cessation. 48% of dual users who made a quit attempt reported ‘trying to stop smoking by exchanging to smokeless tobacco’ [44]. Therefore, the use of an amount of cigarette smoking would be associated with smokeless tobacco by age and attempt to quit smoking.

Although the WHO Framework Convention for Tobacco Control (FCTC) had ratified, the worldwide focus has been mainly on cigarette consumption and with little progress on smokeless tobacco prevention [45]. Starting with Australia in 2012, the plain package of tobacco products was implemented in several countries, including France, the UK, Norway, Ireland, New Zealand, Hungary, Thailand, Uruguay, Slovenia, and Singapore [46]. The pictorial health warnings were implemented on tobacco products including smokeless tobacco products. However, the smokeless tobacco products had generally compact packages at a low price, allowing minors to have high access and negating the influence of tobacco taxation policy. Furthermore, it renders the pictorial health warnings less visible and rather ineffective [47]. Thus, powerful evidence-based cessation policies of smokeless tobacco are needed including products taxation and a ban on advertising and promotion.

This study has several limitations. First, the design of this study was cross-sectional, which may hinder interpreting as a causal relationship. However, this study analyzed three-year pooled data with a nationally representative cross-sectional survey, using propensity scoring matching. Second, since we used national data, it is difficult to quantify the amount of smokeless tobacco use. Some participants might have used more smokeless tobacco products, while others might have used less smokeless tobacco products. Further studies with longitudinal design and mixed-method approaches are needed to gain in-depth information about smokeless tobacco use in young adults. Additionally, it is necessary to check external validation with other countries’ dataset.

Despite these limitations, the study findings add to our knowledge of the association between smokeless tobacco use and cigarette smoking amount by age. In this study, we used three-year pooled data because other smokeless studies used one-year cross-sectional data. Since it might be possible that the characteristics of the case and control groups were different due to bias, we tried to reduce such bias by using propensity scoring matching. This method of analysis could provide multidirectional observations for each user of smokeless tobacco by continuous measurement of the factors that caused the smoking behavior according to the change of status including age [48]. In the case of a causal explanation of the cause of change, these studies according to the flow of time are required rather than a cross-sectional study.

## Conclusions

The present study revealed that among those who were aged under 30, smokeless tobacco use was positively associated with the number of cigarettes used per day. Smokeless tobacco users who were aged under 30 and tried quitting smoking used more cigarettes than those who did non-smokeless tobacco users. Smoking cessation efforts have traditionally concentrated on youth, while marketing to young adults is generally unopposed [49]. Prevention efforts of smokeless tobacco including counter-marketing campaigns should be designed and implemented for young adults to ensure that smokeless tobacco use within this group does not continue to increase. This study could contribute to understand the behaviors and tendencies of smoking in young adulthood and to establish effective smoking cessation methods for their age.

## Data Availability

The datasets generated and/or analysed during the current study are available in the National Center for Health Statistics in Center for Disease Control and Prevention repository, https://www.cdc.gov/nchs/nhis/nhis_2017_data_release.htm.
